# Supplementary material for: Unveiling Chemical Cues of Insect-Tree and Insect-Insect Interactions for the Eucalyptus Weevil and Its Egg Parasitoid by Multidimensional Gas Chromatographic Methods
Source: Molecules. 2022 Jun 23;27(13):4042. doi: 10.3390/molecules27134042 (PMC9268296; doi:10.3390/molecules27134042)
Supplement: Supplementary file 1 [file molecules-27-04042-s001.zip › molecules-1766131-supplementary.pdf]

Supplementary Materials

Unveiling Chemical Cues of Insect-Tree and Insect-Insect Interactions for the Eucalyptus Weevil and Its Egg Parasitoid by Multidimensional Gas Chromatographic Methods

Davide Mendes [1], Sofia Branco [2,3,*], Maria Rosa Paiva [3], Stefan Schütz [4], Eduardo P. Mateus [3] and Marco Gomes da Silva [1,*]

[1] Associated Laboratory for Green Chemistry (LAQV) of the Network of Chemistry and Technology (REQUIMTE), Chemistry Department, NOVA School of Science and Technology, NOVA University of Lisbon, 2829-516 Caparica, Portugal; dm.mendes@campus.fct.unl.pt

[2] The Forest Research Centre (CEF), School of Agriculture University of Lisbon (ISA), Tapada da Ajuda, 1349-017 Lisbon, Portugal

[3] Center for Environmental and Sustainability Research (CENSE), Department of Environmental Sciences and Engineering, NOVA School of Science and Technology, NOVA University of Lisbon, 2829-516, Caparica, Portugal; mrp@fct.unl.pt (M.R.P.); epm@fct.unl.pt (E.P.M.)

[4] Department of Forest Zoology and Forest Conservation, Buesgen−Institute, Göttingen University, 37077 Göttingen, Germany; s.schuetz_137@gmx.de

* Correspondence: sofbranco@hotmail.com (S.B.); mdr@fct.unl.pt (M.G.d.S.)

Supplementary Figures

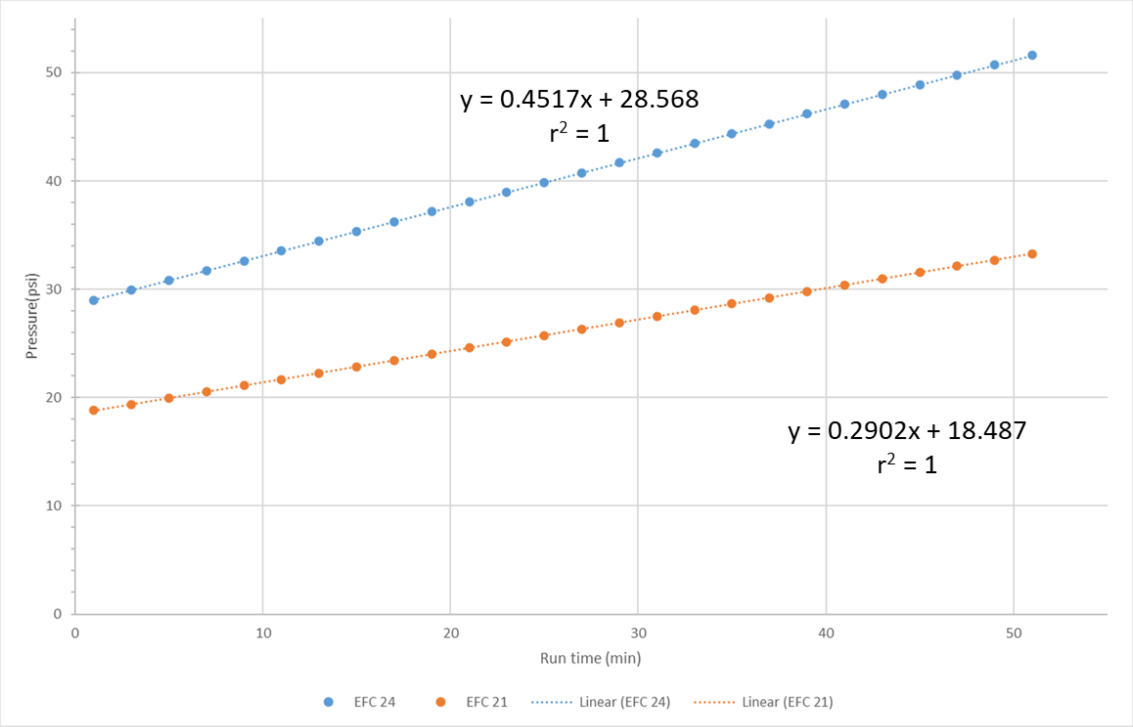

Figure S1. Plot of pressure variation at constant flow during a chromatographic run: EFC 21 at 2.0 mL·min^{-1} and EFC 24 at 1.9 mL·min^{-1}.

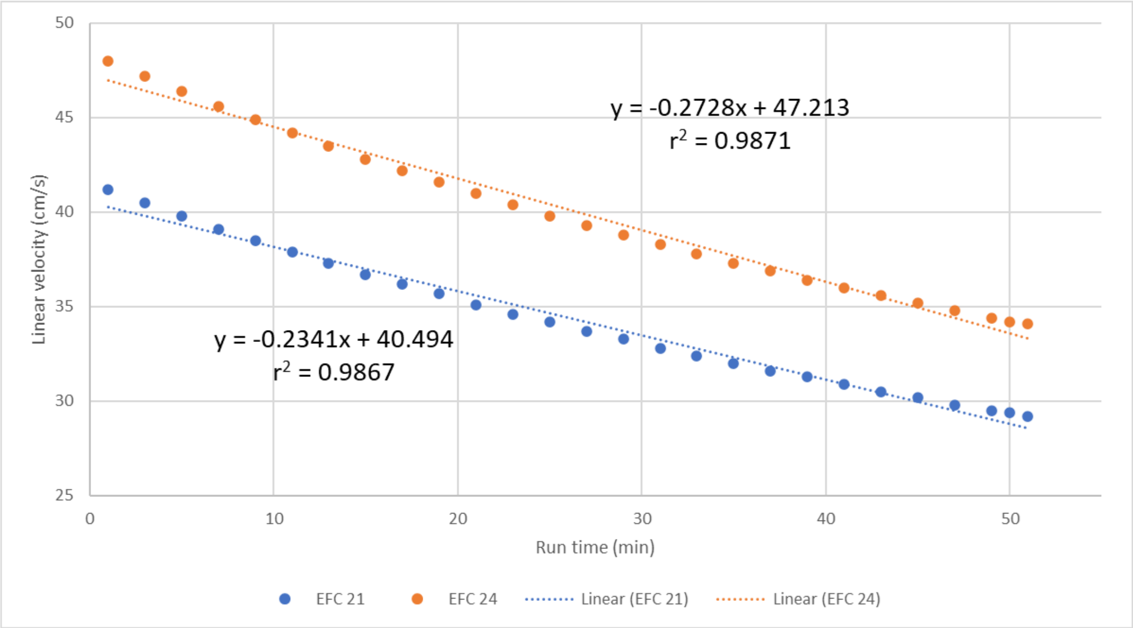

Figure S2. Plot of linear velocity variation of the carrier gas at constant pressure, 35 psi at EFC 21 and 23 psi at EFC 24, during a chromatographic run.

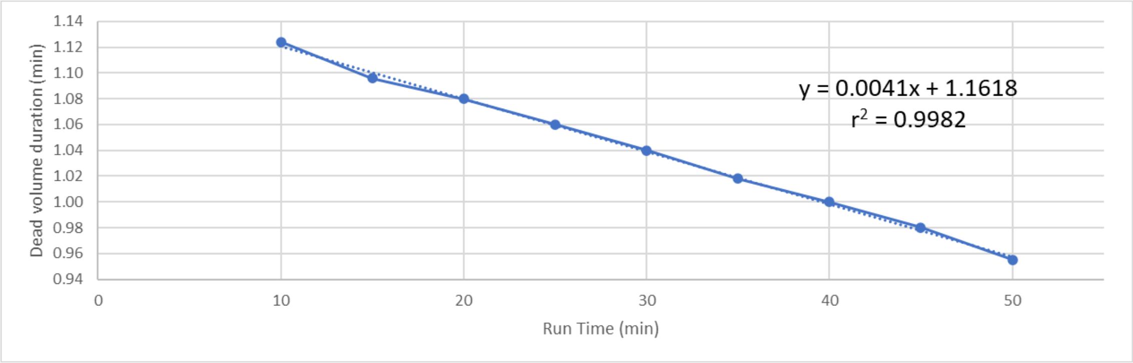

Figure S3. Plot of dead volume time variation in CF mode (EFC 21 at 2.0 mL·min^{-1} and EFC 24 at 1.9 mL·min^{-1}.), during a chromatographic run.

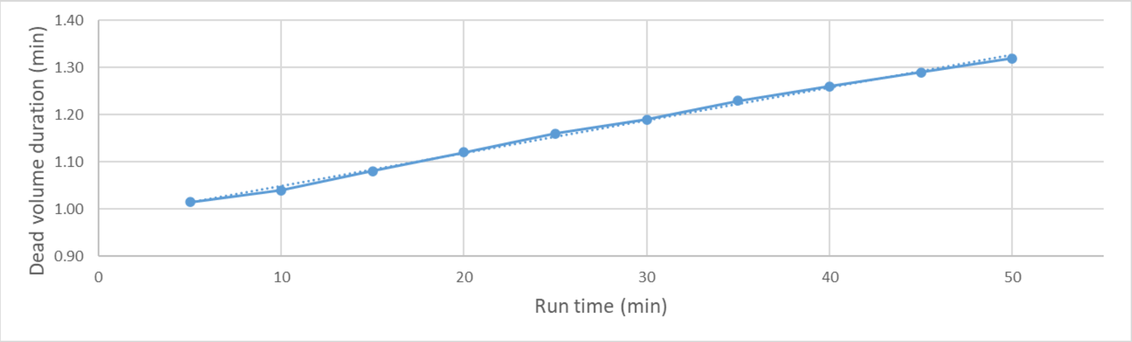

Figure S4. Plot of dead volume time variation in CP mode (35 psi at EFC 21 and 23 psi at EFC 24) during a chromatographic run.

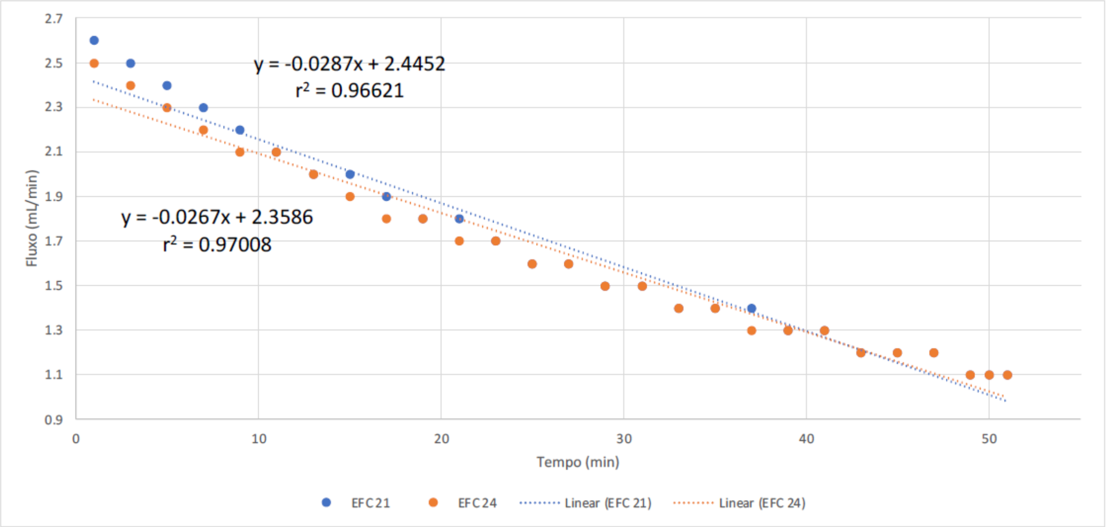

Figure S5. Plot of flow variation of the carrier gas at constant pressure, 35 psi at EFC 21 and 23 psi at EFC 24, during a chromatographic run

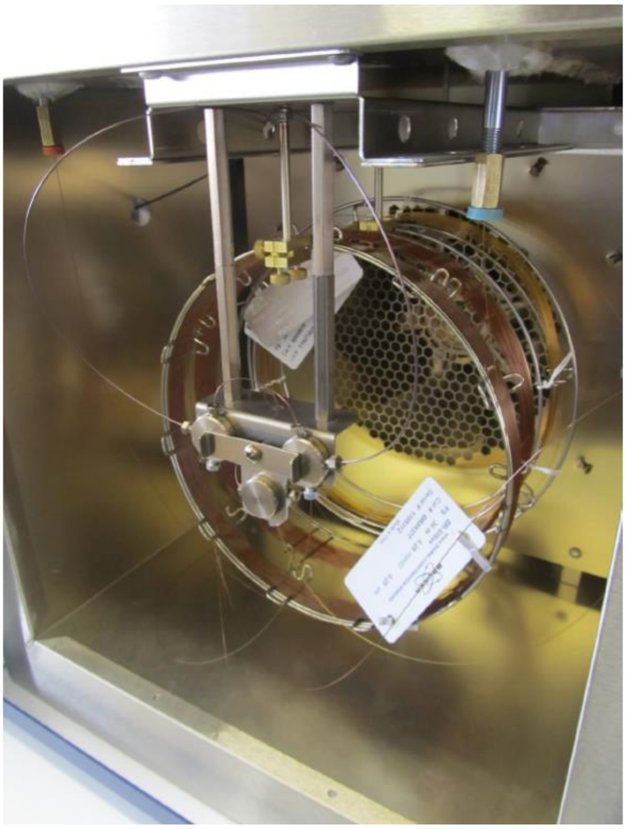

Figure S6. Dean Switch and columns set-up inside the oven.

Supplementary Table

Table S1. *r* values obtained between pairs of male emitted compounds for the three day sampling period.

Compound/ Pearson r	Verbenene	*trans*-Pinocarveol	*cis*-Verbenol	*trans*-Verbenol	3-Oxo-1,8-cineole	Verbenone	2-Oxo-1,8-cineole	2-α-Hydroxy-1,8-cineole	a-3-Hydroxy-1,8-cineole	7-Hydroxy-1,8-cineole	9-Hydroxy-1,8-cineole	Myrtenol
Verbenene	1.00	**0.97**	**0.92**	**0.97**	0.39	0.82	0.51	0.72	0.17	0.65	0.63	**0.95**
trans-Pinocarveol		1.00	**0.90**	**0.98**	0.35	0.84	0.45	0.75	0.14	0.61	0.63	**0.96**
cis-Verbenol			1.00	**0.90**	0.58	0.75	0.64	0.83	0.20	0.20	0.75	**0.93**
trans-Verbenol				1.00	0.28	0.82	0.42	0.69	0.14	0.62	0.61	**0.95**
3-Oxo-1,8-cineole					1.00	0.32	0.75	0.66	0.08	0.35	0.56	0.48
Verbenone						1.00	0.59	0.60	0.01	0.44	0.51	0.89
2-Oxo-1,8-cineole							1.00	0.50	0.06	0.28	0.49	0.57
2-α-Hydroxy-1,8-cineole								1.00	0.33	0.73	0.86	0.79
a-3-Hydroxy-1,8-cineole									1.00	0.42	0.35	0.14
7-Hydroxy-1,8-cineole										1.00	0.84	0.65
9-Hydroxy-1,8-cineole											1.00	0.67
Myrtenol												1.00
